# Experiences with physical activity, health and well-being among young adults with serious mental illness

**DOI:** 10.1080/17482631.2023.2221911

**Published:** 2023-06-10

**Authors:** Jan Freddy Hovland, Eva Langeland, Ottar Ness, Bente O. Skogvang

**Affiliations:** a Department of Public Health and Sport Sciences, Faculty of Social and Health Sciences, Inland Norway University of Applied Sciences, Elverum, Norway; b Vestfold Hospital Trust, Division of Mental Health & Addiction, Tønsberg, Norway; c Department of Health and Caring Science, Faculty of Health and Social Sciences, Western Norway University of Applied Sciences, Bergen, Norway; d Department of Education and Lifelong Learning, Norwegian University of Science and Technology, Trondheim, Norway

**Keywords:** Mental illness, physical activity, qualitative research, recovery, salutogenesis

## Abstract

**Purpose:**

To explore how young adults with serious mental illness (SMI) experience physical activity and how these experiences influence their perceived health and well-being.

**Methods:**

Nine young adults with SMI who had participated in an aerobic high-intensity interval training program were interviewed in depth. The interviews were transcribed and subjected to reflexive thematic analysis.

**Results:**

The results indicated that people with SMI mainly experience physical activity as a meaningful activity that contributes to an increased sense of well-being and better health. However, to overcome various barriers, it is crucial to experience social support and encouragement. The following three main themes were identified through reflexive thematic analysis: (1) positive changes in focus and an increase in well-being occur through physical activity; (2) increased mental strength results from physical activity; and (3) a lack of support and feelings of safety prevent physical activity.

**Conclusions:**

This study shows that adapted physical activity is an important resistance resource that can promote stronger self-identity, increased mental well-being and social engagement and thus contribute to an improved ability to manage stressors. Furthermore, the findings reveal that to engage in physical activity and promote sustainable life changes, it is important for individuals to choose a physical activity based on personal interest and meaning.

## Background

Participating in physical activities is important for improving mental health, well-being and quality of life (WHO, 2013, 2019). Good mental health enables better coping with stressors and stimulates increased participation in the community (Keyes, 2014; WHO, 2013, 2019). Røset (2019) emphasized the importance of regular physical activity from a young age and emphasized that physical activity in school during adolescence seems to positively affect social processes and the development of self-identity, self-esteem and self-worth, thus promoting mental health.

Despite this knowledge, physical inactivity is one of the world’s largest public health problems (WHO, 2016), and it has been well documented that people with serious mental illness (SMI), such as schizophrenia, are significantly less physically active and in poorer health condition than the general population (Stubbs et al., 2016). Furthermore, research has shown that the high incidence of physical inactivity in people with SMI can cause a reduction in life expectancy of up to 20% (Lawrence et al., 2013; Saha et al., 2007). In addition, studies have underscored that an increased level of physical activity might directly extend life expectancy (Schmitt et al., 2018; Vancampfort et al., 2015).

Several studies have shown that, in addition to recovery-oriented practises (meaningful everyday life, job, supportive relationships, and treatment), physical activities can be of great help and support for people with SMI (Davidson et al., 2020; Firth et al., 2015; Ness et al., 2014). Improved physical fitness seems to reduce limitations in daily activities and increase quality of life by providing meaningful activity that promotes a sense of mastery and increased hope for the future (Soundy et al., 2015; Soundy, Freeman, et al., 2014a; Vancampfort et al., 2015).

Studies have revealed that a variety of physical activities promote physical and mental health among people with SMI (Firth et al., 2015). Furthermore, Hargreaves et al. (2017) and Rastad et al. (2014) found that physical activity in general led to a reduction in the negative effects of hearing voices, increased appreciative thinking, and provided a sense of feeling more “normal” by being engaged in physical activity. Activities with low to moderate intensity, such as walking, were revealed to be useful for increased social engagement and working through thoughts, and walking in nature reduced the level of perceived stress and helped individuals connect to real life through more realistic and constructive reflections (Hargreaves et al., 2017; Rastad et al. 2014). Furthermore, studies have shown that the therapeutic and restorative benefits of nature and natural environments are particularly rich in the characteristics necessary for restorative experiences (Abraham et al., 2010; Bratland-Sanda et al., 2019; Kaplan, 1995). In addition, the therapeutic effect of nature, such as wilderness therapy, might promote a stronger sense of coherence (SOC) and direction in life in the longer run, as well as improved self-confidence in social interactions (Gabrielsen et al. 2019). More intense activities, such as running, seem to provide distraction since they are physically demanding and create bodily awareness through the experience of pain due to the activity (Hargreaves et al., 2017).

Studies have identified several different barriers that can prevent people from being physically active, and one of the major barriers seems to be having no one with whom to exercise (Firth et al., 2018). In addition to lack of support, social anxiety, the influence of symptoms, low motivation, low self-confidence and anxiety regarding utilizing facilities along with other people have frequently been found in studies (Firth et al., 2016; Soundy, Stubbs, et al., 2014b). Furthermore, it appears that a high level of perceived stress and moodiness and fatigue or lack of energy prevent engagement in physical activity (Firth et al., 2016; Rastad et al. 2014). It has been shown that social support and encouragement are needed to overcome perceived barriers and to be able to perform, participate in and maintain interest in physical activity (Firth et al., 2016; Gross et al., 2016).

To promote engagement and maintenance of interest in physical activities, it seems important that individuals perform activities that give them a sense of mastery, that they experience support and encouragement and that they perform activities that they themselves select (Vancampfort et al., 2015). Experiencing activities as meaningful or pleasurable generally promotes mental health by positively affecting one’s sense of self-identity and social inclusion (Davidson et al., 2020; Dell et al. 2021). Snethen et al. (2021) found that social participation in everyday activities increased physical activity levels and stimulated a more active lifestyle and that increased social participation in meaningful activities was something that people with SMI often wanted. In addition, support in social engagement and participation could positively promote the level of physical activity through increased opportunities for walking. Furthermore, promoting physical activity could be more effective through a focus on everyday activities instead of a strict focus on exercise since exercise might not be of interest to everyone with SMI (Snethen et al., 2021).

Studies have emphasized that most of these different barriers to physical activity can be positively influenced and that qualitative research can be useful in exploring these barriers to identify how to promote and facilitate physical activity in people with SMIs (Gross et al., 2016; Soundy, Stubbs, et al., 2014b). Furthermore, studies have also highlighted the importance of exploring which factors are essential to motivate people with SMIs to engage in physical activity, as well as maintaining the desire to be physically active (Vera-Garcia et al., 2015). However, research on what type of support is valuable and how this support facilitates physical activity engagement in people with SMI remains scarce and must be established (Gross et al., 2016; Soundy, Stubbs, et al., 2014b; Vera-Garcia et al., 2015). In addition, few studies have been conducted in people with SMI from a salutogenic health-promotion perspective (Jormfeldt, 2011; Langeland & Vinje, 2022), and it is emphasized that a salutary perspective is essential to promote flourishing mental health and minimize the risk of mental illness (Keyes, 2007, Keyes, 2014).

Accordingly, the present study, from a salutogenic, health-promoting perspective, explores how people with SMI experience physical activity and the resources that they need to perform and participate in physical activity.

## Theoretical framework

The theory of salutogenesis is a robust resource-oriented and health-promoting theory that proposes health as a dis/ease-ease continuum and focuses on identifying health-promoting factors that contribute to moving people to the healthy (ease) end of the continuum (Antonovsky, 1996; Eriksson & Lindström, 2008). The sense of coherence (SOC) and general resistance resources (GRRs) are the main concepts of the theory and are especially related to mental health (Eriksson & Lindström, 2008; Lindström & Eriksson, 2006). The SOC can be seen as one’s attitude or orientation of views of life and one’s capacity to respond to stressors, as well as one’s ability to identify and use available resistance resources to promote health and well-being (Lindström & Eriksson, 2006). More specifically, SOC expresses the extent to which one experiences 1) stressors as structured and explainable (comprehensibility); 2) the ability to use the resources necessary to cope with stressors (manageability); and 3) stressors as challenges worth engaging in (meaningfulness) (Antonovsky, 1992; Mittelmark et al., 2022). A GRR is defined as “every characteristic of a person, group or environment that promotes effective management of tension” (Antonovsky, 1979, p. 99).

The model posits that when individuals face stressors, the sense of coherence helps to mobilize GRRs to cope with stressors, thus promoting health and well-being (Mittelmark et al., 2022). GRRs comprise resources such as social relationships and support, self-identity, knowledge, cultural stability and physical activity, and their use can enhance comprehensibility, manageability or meaningfulness, which again might promote the management of stressors and the promotion of the sense of coherence (Antonovsky, 1996; Mittelmark et al., 2022). Furthermore, due to a dynamic and reciprocal dependent relationship between the SOC and GRRs, where the other strengthens or weakens, a strong sense of coherence increases the ability to adapt, recognize and activate the most suitable GRRs (Antonovsky, 1979; Mittelmark et al., 2022).

Physical activity is an important GRR and can, similar to other GRRs, be placed on a continuum from a high GRR to a lack of GRR, called general resistance deficit (GRD). Physical activity is usually defined as any bodily movement that results in energy expenditure and can involve activities such as household, occupational, sports and conditioning activities (Haskell et al., 2007). The WHO recommends that adults undertake 150–300 minutes per week of moderate-intensity or 75–150 minutes per week of vigorous-intensity physical activity (Bull et al., 2020). This recommendation can also be achieved by an equivalent combination of moderate-intensity and vigorous-intensity aerobic physical activity. In addition, performing regular muscle-strengthening activity is recommended (Bull et al., 2020).

Moderate intensity refers to activities that will result in a faster heart rate than usual (e.g. fast walking). High intensity is referred to as activities that will result in a much faster heart rate than usual (e.g. running). In the present study, physical activity is defined as both a planned and structured physical activity that is performed to improve or maintain a level of physical fitness, as well as any other type of bodily movement that results in energy expenditure, whether of high, moderate or low intensity (Haskell et al., 2007).

## Methods

### Design

The study used a qualitative design to explore and describe participants’ lived experiences of the promotion of health and well-being by physical activity and the factors that can promote or prevent physical activity. Exploring subjective lived experiences and revealing meaning through a process of understanding and interpretation are the focus of research with a qualitative design (Lindseth & Norberg, 2004, 2021; Van Manen, 2014), and a reflexive thematic analysis was used to make sense of the data material (Braun & Clarke, 2006, 2020).

### The researchers’ position and reflexivity

In qualitative research, it is important that researchers share their views, perspectives, bias, and experiences within the researched area to clarify factors that might affect the research. This approach is crucial to enhance the trustworthiness and rigour of the study (Bradbury-Jones, 2007; Polit & Beck, 2014). This process is one of reflexivity that involves a critical self-evaluation of one’s positionality and the acknowledgement that one’s position can affect the research processes and outcomes (Bradbury-Jones, 2007), meaning that researchers must discuss and reflect upon their position and acknowledge the influence that it might have on the study, including how questions are posed and the interpretation of data (Graneheim et al., 2017). To guarantee the credibility of the present study, we ensured that the participants had relevant knowledge of the phenomena under exploration. The fact that they all had participated in the exercise group in the EPHAPS study indicates that they had relevant experience with the phenomenon under study. Additionally, we performed recursive analysis based on peer discussions and returned to the data several times (Connelly, 2016; Graneheim et al., 2017). Three of the authors individually conducted the reflective thematic analysis, which consisted of recursive and iterative analytical phases, and the findings were jointly discussed several times to ensure the trustworthiness of the data. It was essential to discuss whether the data accurately reflected what the participants said in the interviews to enhance credibility and confirmability (Polit & Beck, 2014). The process of analysis was also thoroughly supplemented with a variety of quotations to illustrate the different realities of the lived experiences to ensure authenticity (Polit & Beck, 2014).

Knowledge about the research field is emphasized as an advantage during both interviews and their interpretations (Kvale & Brinkmann, 2015; Malterud, 2017). All of the authors share an interest in health promotion and factors that can prevent or promote health and well-being. Two of the authors have experience within mental health care: one of the authors has experience as a family therapist and performs research on recovery processes from mental health problems, and one has experience within sociology as well as adapted physical activity. Two of the authors have also previously been part of research focusing on physical activity. The first author has been working in mental health care for several years with young adults with SMI.

During the analysis process, the researchers were aware of how their background and research interest could affect the interpretation of the interviewees and attempted to present the findings as objectively as possible. Since it can be challenging to be simultaneously close to the practice field and conscious of one’s own position and background, we found joint discussions regarding the analysis process and findings useful to help reflect upon the process and focus on the participants’ lived experiences and their descriptions. As part of the process of analysing the transcribed interviews, all interviews were reread several times to determine whether their interpretation was accurate or whether our preunderstanding had interfered with the analysis. Although our analysis seemed accurate, it is important to acknowledge that our preunderstanding might have influenced our analysis process and interpretation of the participants’ descriptions. None of the researchers had any ongoing treatment relationships with any of the participants in the study.

### Recruitment and participants

This study was a part of the Effects of Physical Activity in Psychoses (EPHAPS) study, which explored the potential physical and mental effects of aerobic high-intensity interval training compared to sport simulation computer gaming in people with schizophrenia spectrum disorder (see Andersen et al., 2018; Engh et al., 2015 for further details).

To ensure relevant descriptions and experiences, the participants were recruited by strategic selection (Patton, 2015) from a pool of 83 individuals (of which 72 completed) who had participated in the EPHAPS study; the inclusion criteria were as follows:

- Less than 30 years of age when participating in the intervention;
- Participation in the exercise group (to ensure physical activity experience);
- Considered to be in a relatively stable mental state; and
- Living at home as a resident in the community.

Researchers from EPHAPS were contacted to discuss possible candidates based on experiences from previous interviews in EPHAPS to ensure interest and the ability to provide thorough descriptions of lived experiences. After a joint discussion between the first author and researchers from EPHAPS, 14 possible participants were contacted by telephone and given information about the study and their participation, after which nine (six men and three women) agreed to participate in the study. Five of the participants lived alone in rental or owned housing. Two lived with their parents, and two lived with a partner. Seven received state benefits and were unemployed. One had a regular paying job, and one received state benefits and worked a few hours per day in a municipal work initiative. The participants had been living with SMI (in terms of diagnosis and/or the first psychotic episode) for an average of eleven years. All the participants expressed having experienced difficulties in general function and social relations from early childhood, which they later related to influence from their mental illness. One participant was only 14 years old when diagnosed with mental illness.

In addition to the recommendations made by the researchers from EPHAPS, the participants’ mental state was assessed at the beginning of each meeting. The most important point was that they were considered to have the ability and stability necessary to engage in dialogue. To create a safe atmosphere and make the interview situation comfortable for the participants as well as to observe signs of discomfort or mental disturbance, a total of 30–45 minutes were spent on “small talk” prior to all of the interviews. The participants were also asked directly how they assessed their own current mental status. The contents of the “small talk” ranged from discussions of their experience of participating in the EPHAPS study, their current situation or whether they had done something positive or interesting lately as well as verifying that the aim and type of study was understood.

### Data collection

Data collection was conducted using individual qualitative interviews. Based on the topics of the research questions, a semistructured interview guide was developed to address topics related to experiences of physical activity, barriers to and prerequisites for physical activity, and the influence of physical activity on mental health and well-being. The questions included the following examples: In your own words, how do you experience being physically active? What do you think or experience is important for being or becoming more physically active (type of activity/adaptation of physical activity/socially/support)? Could you possibly describe whether you have had any experiences in which being physically active has contributed positively to your life, and if so, in what way? In what way do you think that physical activity might have contributed to improved coping with your challenges and/or a sense of mastery?

The interviews were conducted between July 2019 and November 2019.

The interviews lasted between 90 and 150 minutes and were conducted and transcribed by the first author. The participants were given the option of holding the interviews at their homes if desired. Alternatively, the participants agreed to participate, and the interviews were conducted on the premises of the Division of Mental Health and Addiction, Vestfold Hospital Trust.

### Ethical considerations

Study approval was given by the Regional Ethics Committee of Southern and Eastern Norway (reference number 2014/372). The study complied with the principles of the Helsinki Declaration and the requirement to exercise caution in the conduct of research with vulnerable groups (World Medical Association, 2018).

It was agreed in advance that the interviewer could contact the participant’s therapist or general practitioner if concerns arose about the participant’s condition and the need for increased follow-up. Thorough information about the study was given, both verbally and in writing, to the participants to ensure informed consent. The participants were informed of the aim of the study, as well as the possible benefits and risks and their ability to withdraw from the study at any point. During the interviews, the interviewer watched for signs that could indicate that the participants did not want to talk about a certain theme or that a theme made the participants uncomfortable. To avoid unnecessary strain, the topic discussed was changed if it was obviously necessary, or if the interviewer was in doubt, the participant was asked if the topic was too difficult. It was not necessary to contact the participant’s therapist or general practitioner for any of the participants.

Details such as names and places have been changed to safeguard the participants’ anonymity and protect their identities. All names used are pseudonyms to ensure the participants’ anonymity.

### Data analysis

Reflexive thematic analysis was performed to explore and understand the participants’ lived experiences (Braun & Clarke, 2006, 2014, 2022). Reflective thematic analysis is a well-established and recommended analytic approach when researching health and well-being (Braun & Clarke, 2014) and is commonly used when researching lived experience and mental health (e.g. Braun et al., 2014; Crowe et al., 2015). The salutogenic lenses applied in the present study contribute to focusing on experiences and factors relevant to health, sense of coherence and GRRs during the analysis process. The following recursive and iterative analysis phases were followed.

1. To become familiar with the data, the orthographically transcribed interviews were read and reread closely to acquire an overview of the participants’ subjective lived experiences, and the authors’ initial thoughts, ideas and themes were noted.
2. Subsequently, initial ideas or meaningful units that were considered relevant to the research question and the salutogenic perspective were identified and initially coded after a thorough review of each interview.
3. Preliminarily coded ideas and seemingly important patterns in the participants’ subjective experiences were identified, condensed, interpreted, labelled and categorized into potential themes (patterns) across the interviewees.
4. Quotes and descriptions of emerging themes that contained meaningful units were then identified and sorted into five tentative main categories (thoughts and feelings about physical activity, prerequisites for being physically active, experiences of mastery and meaning through physical activity, effect on symptoms through physical activity and social factors related to physical activity). NVivo software (version 12) was used to systemize and categorize meaningful units into different themes.
5. Subsequently, to ensure that a focus on the participants’ lived experiences was maintained, the relevance of the potential themes related to external heterogeneity, internal homogeneity and similarity between themes was jointly examined. The main themes and subthemes were identified, and to show differences and coherent relations between the themes and subthemes, a thematic map was generated. The 1^st^ to 5^th^ phases were performed individually by the first, second and fourth authors, and the findings were then discussed in an analysis workshop.
6. All four authors jointly discussed the analysis process and findings to identify similarities and differences in prominent opinion content, themes and subthemes to ensure internal validity and reduce the risk of research bias. All of the authors contributed to writing the text describing the themes and the ensuing discussion.

The discussion resulted in the identification of more accurate themes and subthemes that captured the experiences and descriptions that emerged in the interviews. Table I in the results section provides an overview of the reflexive thematic analysis.

**Table I.** Overview of the reflexive thematic analysis.

Meaningful unit	Codes	Subthemes	Main themes
*“When I go for a long walk with my dog, I feel that the stress and anxiety gradually disappear, and I am starting to feel calmer and more relaxed. Even though I’m so full of anxiety that I almost throw up, I try to focus on the fact that the anxiety disappeared last time I went for a walk, so it will probably disappear this time also… I feel much calmer and like a new person when I return home because then I have gained a sense of mastery.”*	Reducing stressors Feeling positive emotions	Positive focus during physical activity Reconnecting through walking Active adaptation	Positive changes in focus and increased well-being
*“Being physically active gives me a sort of sense of doing something of importance. It gives a positive feeling when you recognise that you are able to run faster than the last time. It feels very positive to see yourself improving. It provides a sort of self-esteem that is much more in line with what I would like to have. In addition, in a way, you experience what it was like before your challenges began.”*	Finding my potential Improving self-esteem	Doing something meaningful Coping with stressors	Increased mental strength
*“Going to the gym can be a bit like … other people. The problem is that there are other people, and it makes me feel insecure about myself. In addition, if I feel that there is too much fuss [if is too complicated]; I kind of get a reluctance to do it.”*	Not feeling safe Needing motivation	Social anxiety Struggling to find motivation	A lack of support and feelings of safety preventing physical activity

## Results

The results from the reflexive thematic analysis regarding experiences of physical activity and factors that promoted or prevented engagement and participation in physical activity among young adults with SMI are presented in Table I. The results are presented following three main themes and their subthemes.

### Positive changes in focus and increased well-being

The participants talked about their experiences of an increased sense of positive emotions due to engagement in some sort of physical activity. Furthermore, physical activities were described as activities that could lead to a reduction in symptoms and problems caused by the illness. The following aspects of this main theme were described: (1) *positive focus during physical activity;* (2) *reconnecting through walking;* and (3) *active adaptation.*

#### Positive focus during physical activity

Several of the participants expressed that intense physical activity such as running somehow forced them to focus on the activity itself rather than their illness and that they often simultaneously experienced a decrease in symptoms, as illustrated by the following statement from Erik:

*When you are really, really exhausted, and there are just ten or a hundred metres left to run, you don’t have the energy to think negatively. You are just there, and everything is focused on what happens just there. You somehow wear out that engine that keeps running in the back of your head, and everything sort of disappears. Every process is being turned off, and it’s kind of nice to get rid of stuff lying on your shoulders, or something like that.*

Frank also experienced changes in focus and decreased symptoms and described that those negative emotions and thoughts disappeared during physical activity:

*When you do some kind of physical activity, you somehow drag the focus towards something else, and the things that felt difficult sort of go in the background or become easier to push away. After a while, you don’t feel irritated; you just feel tired because you’re running. I also feel that I get much more energy and don’t get tired as quickly when I’m more physically active than when I’m not.*

Intense physical activity creates a state of well-being during the activity by focusing on the activity instead of negative thoughts and difficulties.

#### Reconnecting through walking

The participants often did not consider walking to be physically active since it was something that they simply did to get some fresh air, go to an appointment, or go to the grocery store. Walking was described as a sort of physical activity that did not require any preparations and that was not demanding. Walking was consistently described as positive, and the participants expressed that this activity always felt refreshing and helped to reduce the level of stress, making it easier to think more clearly and reconnect to life. The experience of walking as refreshing and providing a sense of increased well-being is exemplified by a statement from Espen:

*“Being able to walk for a while and move your body helps a lot, and you get a good feeling – you really do. Knowing that you aren’t just being indoors feels good.*

*Being able to walk is very refreshing – to feel that you’re using your body.*

*You are able to see things - experiencing something new and also clear your head a bit. Being able to get some privacy and in a way just exist.”*

Both Anne and Frank described similar experiences of feeling that their stress disappeared when they were walking, and they experienced improved self-esteem and other positive effects due to walking:

*There are a lot of nice things to see when you are outside walking. You get fresh air, and you are also getting tired in a good way after a long walk. And it feels good knowing that you have been outside walking instead of just sitting on the couch all day [Ann].*

Frank illustrates that when moving the body, thoughts and feelings also move:

*“When you are out walking, you are just focusing on nature or the person you’re walking with. It makes you focus on something other than [your difficulties]….*

*just focusing on something else. You manage to focus on the positive things in life instead of all the crap that is going on.”*

Walking provides opportunities for enjoying nature and social interactions and creates positive emotions by knowing that one has done something positive for one’s health.

#### Active adaptation

To avoid mental overload when feeling too influenced by symptoms or feeling sad, the participants sometimes found it necessary to adapt the activity since demanding physical activity, such as boxing or lifting weights, could lead to aggravation. These experiences were illustrated by Siri, who normally lifted weights or performed cardio exercise every day:

*If I’m having a bad day or feeling mentally very tired and push myself a little too much physically, I can become totally worn out because then the total load somehow becomes too much. However, if I’m just a little tired or having just a little worse day than average, exercise can work really well and have a great effect.*

Not being able to exercise due to negative experiences, such as worsening of symptoms, was also described by Per, who exercised for several hours in the gym every day. Normally, he experienced only positive effects when exercising, such as focusing on something other than his illness and challenges in life. However, the days when he felt too influenced by symptoms, he found boxing or lifting weights to be almost impossible:

*Exercising gives me something else to focus on, my physical shape and stuff like that. It gives me something else to focus on, at least while exercising and especially during intense exercise, such as boxing. You get such a nice feeling when doing cardio-exercise. Lifting weights also helps, but in another way. However, if I’m having a bad day, exercising is not possible. I have tried, but it just feels negative. I just have to stay home, relax and wait.*

The participants generally experienced physical activity as positive and enabling a focus on the good things in life instead of the difficult things. Furthermore, physical activities were experienced as activities that helped to decrease symptoms and promote coping with challenges and thus a sort of active participation in promoting one’s health and well-being. However, the need to actively adapt and not try pushing oneself too hard when being influenced by symptoms or feeling depressed was emphasized by the participants since pushing too hard could worsen their situation.

### Increased mental strength

The participants emphasized how they observed that being physically active positively affected their self-perception and provided a sense of doing something positive and meaningful for themselves. Being physically active was described as leading to increased self-confidence and self-esteem, as well as providing a sense of mastery and increased well-being. The aspects of this main theme included (1) *doing something meaningful* and (2) *coping with stressors.*

#### Doing something meaningful

All of the participants described physical activity as something positive and meaningful since they knew and felt that they were actively doing something of importance for their own health and well-being. The ability of physical activity to create more positive thinking, increased well-being and greater interest in life as well as a sense of actively promoting one’s own health is exemplified by the following statement from Ole:

*It gives me a comfortable feeling and helps me understand that there is something more to life [than sitting in his apartment]. It helps with my depression, and it helps me sleep properly. I feel like I’m actually doing something and can put my depression on the shelf, which then makes me experience things less negatively. And I also gain better structure when I exercise, such as when I get out of bed and what time to exercise. I manage to keep track of time.*

Similar experiences of being physically active were also expressed by Per, who had gained better self-confidence and a better self-image, as well as a sense of increased well-being and mastery:

*I feel safer and more self-confident through being physically active. I see that I have good genes and gain muscles, and it makes me feel better about myself. You become more satisfied when you improve your physical condition and experience being in better shape and know that you are able to lift heavier weights than before. Knowing that I did my best that day [when exercising] is also very satisfying, and it gives me a sense of mastery.*

Erik also described a similar experience of physical activity positively affecting his self-perception because he was doing something of importance, and he reported increased well-being through more positive emotions and attitudes towards himself, as well as experiences of meaningfulness:

*Being physically active gives me a sort of sense of doing something of importance. It gives a positive feeling when you recognise that you are able to run faster than the last time. It feels very positive to see yourself improving. It provides a sort of self-esteem that is much more in line with what I would like to have. In addition, in a way, you experience what it was like before your challenges began.*

Physical activity promotes a more positive self-perception by creating structure, meaningfulness, mastery and a feeling of doing something that has a purpose.

#### Coping with stressors

Several of the participants talked about how they went for walks or sometimes just did some push-ups to feel better and attempted to reduce stress by performing some type of physical activity. For example, Trond, who previously had participated in a walking group, said that walking gave him the necessary energy and initiative to do other things, e.g. cleaning the house or taking a shower, which then made him feel better about himself. However, he emphasized that he often struggled to find the strength and initiative to go for a walk on his own and that support through a walking group or going with a friend was useful:

*Walking feels really good, and I enjoy going for a walk. I prefer walking in places where there are few people, like in the forest. Walking gives me the energy to do housework, take a shower or other things that feel difficult to do. If I just manage to get out of the house, I also manage to go for a walk. I* *can* *manage to walk by myself, but I feel much safer when I am with someone due my fear of animals and stuff.*

Both Eva and Ann described that they actively used walking as a way to reduce frustration, anxiety and stress since they had several experiences of beneficial effects through being physically active. Eva had a dog that needed exercise, and she therefore went for walks with her dog several times each day. She expressed her experiences as follows:

*When I go for a long walk with my dog, I feel that the stress and anxiety gradually disappear, and I am starting to feel calmer and more relaxed. Even though I’m so full of anxiety that I almost throw up, I try to focus on the fact that the anxiety disappeared last time I went for a walk, so it will probably disappear this time also… I feel much calmer and like a new person when I return home because then I have gained a sense of mastery.*

Siri explained that she tried to perform some sort of physical activity every day and that she actively used physical activity to reduce stress, being able to think more positively and generally feel better about herself. She also emphasized that it often did not require much effort to achieve a sort of mental and physical well-being:

*You just need to do something that requires some effort and strength – increase the pulse a bit. Suddenly, something happens in your head, and you actually feel happier, and your entire body feels better.*

The participants described experiencing positive emotions, an increased sense of mastery and feeling good about themselves during and after physical activity. Some preferred going to a gym, while others enjoyed going for a walk in their neighbourhoods or in nature. Others again went walking with other people, and some underscored the importance of having a dog or a pet that needed physical activity. By managing to do some sort of physical activity, they felt more self-confident and felt that they had done something of importance for themselves. Several emphasized that they actively used physical activity as a way to reduce stress and felt better by experiencing mastery and increased comprehensibility.

### A lack of support and feelings of safety preventing physical activitya

Most of the participants expressed that they wanted to become more physically active and that being physically active gave them increased self-confidence and self-esteem. However, several of the participants emphasized that they experienced barriers that could prevent them from, for example, exercising in a gym and finding the strength and motivation to overcome these barriers. The following aspects of this main theme were described: (1) *social anxiety* and (2) *struggling to find motivation.*

#### Social anxiety

Insecurity regarding other people, as well as the fear of being judged or looked at with disdain, were described as prominent factors that could prevent the participants from going to a gym. Furthermore, low self-confidence, low self-esteem and negative self-image were also described as major barriers that prevented them from going to a gym, as exemplified by the following description from Ann:

*I feel that people are scary, and I feel that going to a gym is really scary and disgusting. I sense how they look at me and what they think about me. However, if I went there together with somebody that I knew, it wouldn’t be as scary.*

Going to a gym was also described as very challenging by Siri, although she enjoyed being active and doing different types of physical activity. She expressed that the exercises needed to be easy to perform and emphasized that she quickly lost interest and motivation if it was too complicated and involved a risk of failure and hence a possibility of negative attention or being looked at with distain: Siri expressed the barriers preventing her from going to a gym as follows:

*The problem is that there are other people, and it makes me feel insecure about myself. In addition, if I feel that there is too much fuss [if it is too complicated], I kind of get a reluctance to do it.*

Per also described a sense of insecurity and often not feeling completely safe when he started going to the gym. However, this situation changed after a short while when he felt accepted and included, as he experienced that people were friendly and interested in him and stressed that he valued the accepting social climate at the gym:

*It is nice to see and meet people at the gym. Having small talks or just to say hello… Get a smile and give a smile back, it is really, really nice … Sort of getting confirmations of being seen.*

Mastering the activity and receiving support and micro-affirmations from others promote overcoming one’s insecurity and create a stronger interest in continuing physical activity.

#### Struggling to find motivation

Several of the participants described struggling to find the necessary motivation to exercise or become more physically active. Some also expressed that it often could be difficult to see the meaning of engaging in physical activity and emphasized that they did not have the necessary initiative and motivation without receiving support from others. A statement from Ann might illustrate this experience:

*I don’t manage to exercise on my own, and if I had been going to a gym by myself, it would have been completely nonsense. I need someone to push me and kick me in the ass, so to speak.*

Ole also described difficulties in finding the motivation and initiative to become more physically active on his own and underscored that he needed support from others to gain motivation and engagement to exercise, as well as to maintain engagement. He expressed this need for support as follows:

*“It’s not something that I start doing on my own. It’s nice to have someone I can relate to when I am exercising, for example, someone whom I can exercise with to stay motivated. Then, I can also get confirmation about my progress and what I do well.*

*I think I become more satisfied and self-confident knowing that I have actually done something* *– in a way – and I think that’s exactly the kind of motivation that I need.”*

Difficulties in finding motivation to become more physically active and the need for support were also described by Espen, who used to practise karate when he was younger:

*I have often thought about starting to practise karate again because I used to enjoy practising karate. I don’t quite know what stops me from going back. It would be nice if I could have a more definite opinion about what is stopping me or find a reason to start again. It needs to be interesting and also something that I want to do. Perhaps if a friend goes there or something…*

In addition to low motivation or a lack thereof, the participants often struggled with negative self-perception, which led to low self-confidence, low self-esteem and negative self-image. These challenges were described as barriers that made it difficult to engage in physical activity and were often related to social insecurity, such as feeling exposed if exercising in a gym and fearing being judged or looked at with disdain by others. Going with a friend or someone familiar, performing activities that one finds interesting and receiving positive feedback were emphasized as key factors that could provide the necessary feeling of security and motivation to engage in physical activity.

## Discussion

The present study used a salutogenic health promotion perspective to explore how young adults with SMI experienced physical activity, including factors that promote or prevent physical activity.

The results show that physical activity might be an important resource by helping to transform tension and stress into coping, thus possibly strengthening the SOC, health and well-being (Antonovsky, 1987; 1996). Furthermore, the study reveals that physical activity must be experienced as a meaningful activity by being actively adapted so that the person perceives it as easy to perform and based on his or her own choice. In addition, the findings indicate that physical activity leads to experiencing mastery, better self-esteem and greater self-confidence and thus possibly a stronger self-identity. Moreover, physical activity gives one a sense of actively doing something of importance to gain better health and simultaneously being able to influence one’s own life and manage stressors. Our results support the WHO’s (2013, 2019) emphasis that physical activity improves mental health, well-being, and coping with stressors. Additionally, activities experienced as meaningful might promote a stronger motivation that again might strengthen the sense of coherence and willingness to use GRRs (Antonovsky, 1996). However, different barriers can prevent engagement in physical activity and lead to inactivity. The results show that different barriers might be overcome when people are supported and encouraged by others to participate in physical activity. Based on the study results and purpose, we discuss the following issues: (1) *adapted physical activity as an important resistance resource for young adults with SMI* and (2) *social support promoting meaningful physical activity and a more constructive self-identity.*

### Adapted physical activity as an important resistance resource for young adults with SMI

The participants emphasized that adapted physical activity felt meaningful, contributed to reductions in symptoms and led to increased self-reflection and paying of attention to the environment (something outside themselves), rather than a focus on their illness or difficulties in life. Furthermore, appropriate physical activity promoted other important resistance resources, such as mastery, self-esteem, self-confidence and thus self-identity and SOC (Antonovsky, 1979, Langeland & Vinje, 2022).

The present study results support previous research by Firth et al. (2015), Soundy et al. (2015) and Vancampfort et al. (2015) showing that physical activity promotes health and well-being by promoting mastery, reduces limitations in daily activities and increases quality of life. The present study also confirms the findings of Hargreaves et al. (2017) and Rastad et al. (2014) that less intense physical activity can reduce symptoms by creating positive emotions and promoting relaxation and positive awareness of the environment, whereas intense physical activity can reduce various symptoms because intense physical activity demands focus (Hargreaves et al., 2017). Since engagement in physical activity seems to promote life mastery and better self-esteem and self-confidence, it is also likely that this practice creates a positive reciprocal relationship between the use of physical activity as a GRR and experiences of load balance, thus promoting a sense of coherence (Antonovsky, 1996).

The results show that, for some participants, previous positive experiences of physical activity can lead to the active use of physical activity to manage stressors, while other participants found it more challenging to adapt using more suitable physical activity to reduce stress and create load balance. From a salutogenic perspective, having the skills to identify, use and reuse the most suitable GRRs (e.g. walking or push-ups) to cope with stressors indicates a stronger SOC and thus promotes movement to the health (ease) end of the health continuum (Antonovsky, 1979; 1996; Mittelmark et al., 2022). Moreover, the ability to use and learn from positive life experiences and actively adapt is crucial for creating load balance and coping with stressors, thus possibly strengthening the sense of coherence (Antonovsky, 1979; Langeland et al. 2016).

The results show that walking was a kind of physical activity that was easy to perform, provided opportunities for social interactions (walking and talking) and contributed to a sense of increased meaningfulness, which is the most important dimension in the SOC. Moreover, walking transformed stress into coping by increasing awareness of the environment by changing negative focus and helping individuals to reconnect to life. Furthermore, walking was experienced as contributing to achieving a sense of mastery by doing something positive for oneself by engaging in physical activity and contributing to increased motivation to do other things. The results support the findings described in the studies of Hargreaves et al. (2017) and Rastad et al. (2014), who showed that walking contributed to social engagement, helped people reconnect to reality, reduced stress and provided a feeling of being content and pleased with oneself. In addition, walking is also recommended as a type of physical activity that can provide health benefits when performed on a regular basis, and by gradually increasing the duration and intensity, one can achieve the recommended level of physical activity (WHO, 2019). Furthermore, walking has also been found to be a type of physical activity that the majority of people with SMI often prefer (Rastad et al. 2014). The present study shows that walking in nature and natural environments might be an important GRR by creating the experiences of belonging to something larger, greater calmness and a more constructive mindset. This outcome is compatible with the findings of Abraham et al. (2010), Bratland-Sanda et al. (2019) and Kaplan (1995) of how walking in nature and natural environments creates positive experiences that can have therapeutic and restorative benefits and possibly promote a stronger SOC (Gabrielsen et al. 2019).

### Social support promotes meaningful physical activity and a more constructive self-identity

As shown in the results, different barriers (GRDs), such as social insecurity, negative self-image, low self-confidence and influences of symptoms, could prevent participation in physical activity, such as going to a gym. Similar barriers were found in the studies of Soundy et al. (2014b), Firth et al. (2016) and Rastad et al. (2014), which showed that people with SMI can experience numerous barriers preventing physical activity. However, the results from our study show that these barriers can be overcome or experienced as less challenging in the presence of social support, such as having someone to exercise with in a gym or on a walk. These findings are consistent with those of Firth et al. (2016) and Gross et al. (2016), who emphasized that experiencing social support is of great importance for engaging in and maintaining physical activity. A lack of social support (i.e. a training partner) was also emphasized by Firth et al. (2018) as a major barrier to being physically active.

The study by Snethen et al. (2021) showed that experiencing social support can promote increased social participation in everyday activities and a higher level of physical activity through increased opportunities for walking. Additionally, promoting everyday activities can be an effective way to help people become more physically active since not everyone with SMI finds physical activity through exercising interesting (Snethen et al., 2021). The present study shows that physical activities, such as walking, running or lifting weights, were experienced as positive activities that felt meaningful to engage in and were perceived to contribute to a sense of active participation in one’s own life, thus promoting better SOC, mental health and well-being. This outcome might underscore the findings from Snethen et al. (2021), who noted that one must seek activities of interest to oneself to create active substantial life changes, which was also emphasized by Lundström et al. (2017). The importance of experiencing activities as positive and meaningful was emphasized by Davidson et al. (2006), Davidson et al. (2020) and Dell et al. (2021) since such experiences promote health and well-being and hence promote recovery by positively affecting one’s sense of social identification and self-identity. From a salutogenic perspective, activities that create good feelings and motivation are crucial factors for promoting the meaningfulness component of the SOC. Accordingly, positive experiences of physical activity might promote the SOC and thus stimulate perceptions of stressors as worth facing and provide the energy to search for possible resources to manage the situation (Antonovsky, 1979).

The present study reveals that quality of social support, meaningful activities and self-identity are crucial GRRs. The reciprocal interplay between these GRRs seems to be important for promoting SOC and thus increasing well-being.

### Strengths and limitations of the study

The sample consisted of only nine persons, which might have limited the variety of descriptions and experiences. It is possible that a larger sample would have provided richer and more nuanced findings due to different life experiences and denser descriptions.

However, relevant experience, thorough and various accounts of different aspects of the phenomenon explored, a strong dialogue between the researcher and the participant and the use of an established theory (the theory of salutogenesis) indicate high “information power” and thus a sufficient number of participants (Malterud et al., 2016, 2021).

Although some of the interview questions might seem slightly governing, this choice was intentional. From a salutogenic perspective, it is essential to facilitate the participants in reflecting on how and to what degree they experienced being physically active as a health-promoting factor (GRR) and thus created knowledge about salutogenesis in a new setting. Accordingly, the focus is on processes that might be health-promoting. Furthermore, the interview guide was also developed in collaboration with three participants from the control group in the EPHAPS study, and the questions aimed to be relevant and easy to understand, as well as to invite discussion and reflection. The fact that several of the participants had met the interviewer in the EPHAPS study might have influenced the interviews both positively and negatively. However, it is reasonable to think that this influence promoted an increased sense of safety and thus created a good climate for sharing experiences and perspectives. At the same time, it is also possible that this relationship prevented the participants from sharing personal thoughts and experiences.

Overall, it is important to describe and investigate people with SMIs experiences respectfully to promote trustworthy knowledge, and as emphasized by Kvale and Brinkmann (2015) and Lindseth and Norberg (2004, 2021), it is essential to create a permissive climate in which participants feel safe and free to share their narratives. It is reasonable to assume that the combination of “small talk” and the interviewer’s experience in mental health care created a positive atmosphere that resulted in vivid and comprehensive descriptions of lived experiences that strengthened the analysis and findings of the study. To assess the extent to which the findings are useful to persons in other settings, this study endeavoured to provide rich and thorough descriptions of the relevant context, participants and analysis.

### Conclusions and implications for practice and future research

As shown by our study, physical activity seems to strongly contribute to the management of stressors and increased mental well-being. It also seems to promote a positive self-identity and social engagement. This study supports previous research (Firth et al., 2015; Soundy et al., 2015; Soundy, Freeman, et al., 2014a; Vancampfort et al., 2015) since it shows that physical activity seems to improve perceived health, well-being and quality of life. Furthermore, our study shows that supporting people in actively adapting and finding an activity that is appropriate for them is of great importance. Walking seems to be an activity that is easier to perform than other activities. It appears that there are fewer barriers that prevent engagement in walking as a physical activity compared to running or going to a studio. Walking seems to strongly contribute to better health and management of stressors in everyday life and to possibly strengthen the ability to use appropriate GRRs in different situations. Our results suggest that physical activity is an important resource for people with SMI since it seems to encourage coping with symptoms and stressors, thus promoting good feelings, stronger identity, and better health and well-being. However, as our study reveals and as underscored by Lundström et al. (2017), it is important that individuals choose an activity based on personal preference to create meaningful sustainable life changes. Our results illustrate the importance of facilitating individual preferences and social support to promote engagement in physical activity and promote recovery. When developing guidelines for treatment within mental health care, our results illustrate that these aspects should be considered. However, there is a need for more research on experiences of physical activity among people with SMI, and additional studies might be needed to explore how people with SMI might be supported in the best possible way in physical activities selected based on their own preferences, thus promoting sustainable life changes. For future research, it would be interesting in a randomized, controlled trial to test the effect of how participation in walking groups (GRRs) promotes increased social participation, greater support and the sense of coherence and thus promotes recovery in people with SMI.
